# Tyrosyl-tRNA sequestration triggers tyrosyl-tRNA synthetase-associated peripheral neuropathy

**DOI:** 10.1093/brain/awag204

**Published:** 2026-08-12

**Authors:** Moushami Mallik, Abigail L D Tadenev, Natalia Mora, Vanessa Lewandowski, Daniel Köster, María Landínez-Macías, Robin Thompson, María P Menafra, Noah K Taylor, Pascal van Lith, Nick van Bakel, Marica Catinozzi, Scott Q Harper, Zoya Ignatova, Robert W Burgess, Erik Storkebaum

**Affiliations:** Molecular Neurobiology Laboratory, Donders Institute for Brain, Cognition and Behaviour and Faculty of Science, Radboud University, Nijmegen 6525 AJ, The Netherlands; The Jackson Laboratory, Bar Harbor, ME 04609, USA; Molecular Neurobiology Laboratory, Donders Institute for Brain, Cognition and Behaviour and Faculty of Science, Radboud University, Nijmegen 6525 AJ, The Netherlands; Biochemistry and Molecular Biology, Department of Chemistry, University of Hamburg, Hamburg 20146, Germany; Biochemistry and Molecular Biology, Department of Chemistry, University of Hamburg, Hamburg 20146, Germany; Molecular Neurobiology Laboratory, Donders Institute for Brain, Cognition and Behaviour and Faculty of Science, Radboud University, Nijmegen 6525 AJ, The Netherlands; Biochemistry and Molecular Biology, Department of Chemistry, University of Hamburg, Hamburg 20146, Germany; Molecular Neurobiology Laboratory, Donders Institute for Brain, Cognition and Behaviour and Faculty of Science, Radboud University, Nijmegen 6525 AJ, The Netherlands; Jerry R. Mendell Center for Gene Therapy, The Abigail Wexner Research Institute at Nationwide Children’s Hospital, Columbus, OH 43202, USA; Molecular Neurobiology Laboratory, Donders Institute for Brain, Cognition and Behaviour and Faculty of Science, Radboud University, Nijmegen 6525 AJ, The Netherlands; Molecular Neurobiology Laboratory, Donders Institute for Brain, Cognition and Behaviour and Faculty of Science, Radboud University, Nijmegen 6525 AJ, The Netherlands; Molecular Neurobiology Laboratory, Donders Institute for Brain, Cognition and Behaviour and Faculty of Science, Radboud University, Nijmegen 6525 AJ, The Netherlands; Jerry R. Mendell Center for Gene Therapy, The Abigail Wexner Research Institute at Nationwide Children’s Hospital, Columbus, OH 43202, USA; Department of Pediatrics, The Ohio State University College of Medicine, Columbus, OH 43201, USA; Biochemistry and Molecular Biology, Department of Chemistry, University of Hamburg, Hamburg 20146, Germany; The Jackson Laboratory, Bar Harbor, ME 04609, USA; Molecular Neurobiology Laboratory, Donders Institute for Brain, Cognition and Behaviour and Faculty of Science, Radboud University, Nijmegen 6525 AJ, The Netherlands

**Keywords:** Charcot–Marie–Tooth disease, peripheral neuropathy, aminoacyl-tRNA synthetase, molecular mechanism, gene therapy, integrated stress response

## Abstract

Mono-allelic mutations in seven cytosolic aminoacyl-tRNA synthetase genes cause Charcot–Marie–Tooth (CMT) peripheral neuropathy.

Here, we report that tyrosyl-tRNA (tRNA^Tyr^) sequestration by CMT-mutant tyrosyl-tRNA synthetase (TyrRS) is the molecular root cause of CMT-*YARS1*. tRNA^Tyr^ sequestration depletes the cellular free tRNA^Tyr^ pool, leading to insufficient tyrosyl-tRNA^Tyr^ production and ribosome stalling at tyrosine codons.

In three *Drosophila* CMT-*YARS1* models, transgenic overexpression of tRNA^Tyr^ rescued peripheral neuropathy phenotypes and defective mRNA translation. We provide biochemical evidence for tRNA^Tyr^ sequestration by the three CMT-TyrRS variants expressed in the *Drosophila* models and ribosome profiling revealed selective ribosome pausing at the two tyrosine codons. In the *Yars1^E196K^* mouse model, adeno-associated virus serotype 9 (AAV9)-mediated overexpression of tRNA^Tyr^—but not wild-type TyrRS protein—fully rescued motor performance deficits, reduced nerve conduction velocity and reduced motor axon calibre. Furthermore, AAV9-tRNA^Tyr^ gene therapy prevented activation of the integrated stress response in spinal motor neurons, indicating resolution of ribosome stalling.

Thus, elevating tRNA^Tyr^ levels constitutes a novel therapeutic approach for CMT-*YARS1*.

## Teaser

Mallik *et al.* discovered that peripheral neuropathy caused by mutations in *YARS1* arises when mutant YARS1 protein sequesters a vital transfer RNA (tRNA), thus hampering protein biosynthesis. Overexpressing this tRNA in animal models rescued nerve and motor defects, identifying a promising gene therapy strategy.

**See Sleigh (https://doi.org/10.1093/brain/awag340) for a scientific commentary on this article.**

## Introduction

Charcot–Marie–Tooth (CMT) peripheral neuropathy is characterized by length-dependent degeneration of peripheral motor and sensory axons, leading to progressive distal muscle weakness and wasting, steppage gait, sensory dysfunction and often foot deformities that require orthopaedic surgery. A distinction is made between demyelinating forms of CMT (CMT1), axonal forms (CMT2) and intermediate forms which display hallmarks of both demyelination and axonal degeneration.^1,2^ Currently, there is no effective, disease-modifying therapy available for any form of CMT.

CMT is a genetic disease, and mutations in more than 100 genes have been linked to CMT.^3^ Remarkably, dominantly inherited mutations in seven cytosolic aminoacyl-tRNA synthetases (aaRSs) cause axonal and intermediate forms of CMT.^4,5^ aaRSs covalently link amino acids to their cognate tRNA, a reaction termed aminoacylation.^6,7^ Aminoacylated tRNAs are subsequently used by the ribosome to decode mRNA codons into polypeptides.^8,9^

We previously reported that the expression of each of three CMT-associated glycyl-tRNA synthetase (GlyRS) variants or three tyrosyl-tRNA synthetase (TyrRS) variants in *Drosophila* motor or sensory neurons substantially inhibits mRNA translation, indicating that defective protein synthesis may constitute a unifying pathogenic mechanism underlying CMT-aaRS.^10^ Recently, we identified the molecular mechanism underlying CMT-*GARS1* (CMT2D): CMT-GlyRS variants are still able to bind glycyl-tRNA (tRNA^Gly^) but either fail to release it or release at a very slow pace.^11,12^ This tRNA^Gly^ sequestration depletes the cellular free tRNA^Gly^ pool, leaving insufficient substrate for GlyRS derived from the wild-type (WT) *GARS1* allele in heterozygous patients, resulting in insufficient glycyl-tRNA^Gly^ production and ribosome stalling at glycine codons.^11,12^ Ribosome stalling in turn triggers activation of the integrated stress response (ISR), selectively in motor and sensory neurons of CMT-*GARS1* mouse models, which contributes to the peripheral neuropathy phenotypes.^13^ Consistent with this mechanism, transgenic overexpression of tRNA^Gly^ rescued peripheral neuropathy and restored protein synthesis in both *Drosophila* and mouse CMT-*GARS1* models,^11,12^ whereas overexpression of WT GlyRS itself did not.^12,14^

These seminal findings raise the question whether a similar pathogenic mechanism—sequestration of the cognate tRNA—may underly the six other forms of CMT-aaRS. For CMT caused by mutations in *YARS1* (dominant intermediate CMT type C or DI-CMTC),^15^ encoding TyrRS, several pathogenic mechanisms have been proposed, including enhanced interaction with the extracellular domain of the brain-derived neurotrophic factor (BDNF) receptor tropomyosin receptor kinase B (TrkB),^16^ defective actin cytoskeleton organization^17^ and transcriptional dysregulation.^18^ However, the possibility that tRNA^Tyr^ sequestration by CMT-mutant TyrRS underlies CMT-*YARS1* is suggested by the substantial inhibition of global protein synthesis induced by expression of CMT-TyrRS variants in *Drosophila* motor and sensory neurons.^10^ This mRNA translation defect is not attributable to a simple loss of tRNA^Tyr^ aminoacylation activity, because the TyrRS-E196K variant does not affect aminoacylation activity,^19,20^ yet strongly inhibits global protein synthesis.^10^ Furthermore, the ISR is selectively activated in motor and sensory neurons of *Yars1^E196K^* mice,^13^ suggesting ribosome stalling in these CMT-affected cell types. Finally, an unusually high fraction of CMT-*YARS1* causative mutations (5 of 7) result in addition of net positive charge to the TyrRS protein, which may promote sequestration of the negatively charged tRNA^Tyr^.^4^ Therefore, we here evaluated whether tRNA^Tyr^ sequestration may underlie CMT-*YARS1*.

## Materials and methods

### *Drosophila* genetics

Flies were housed in temperature-controlled incubators with 12:12 h on/off light cycle at 25°C, and for some experiments at 18°C and 29°C, respectively. *OK371-GAL4* (Bl 26160), *ppk-GAL4* (Bl 32079) and *Actin5C-GAL4* (Bl 4414) were obtained from the Bloomington *Drosophila* Stock Center (Bloomington). The *nSyb-GAL4* driver line^21^ was kindly provided by B. Dickson. The upstream activating sequence (UAS)-TyrRS and UAS-GlyRS lines were previously described.^10^ For imaging of Class IV da sensory neurons, *ppk-GAL4* was recombined with *ppk-CD4-tdGFP.*^22^ For ubiquitous expression of the UAS-TyrRS transgenes from the adult stage onwards, the GAL80 Temporal and Amplified Regulation of Gene Expression Targeting (TARGET) system^23^ was used. Fly crosses were cultured at 18°C (permissive temperature) and 1-day-old adult flies carrying the *tub-GAL80^ts^; tub-GAL4* and UAS-TyrRS transgenes were shifted to 29°C to induce transgene expression.

### Generation of tRNA^Tyr^ transgenic *Drosophila* lines

For the generation of tRNA^Tyr^ transgenic *Drosophila* lines, Gibson assembly was used to generate a construct that contains three copies of a genomic fragment which contains a cluster of three tRNA^Tyr-GTA^ genes (tRNA:Tyr-GTA-1-6, tRNA:Tyr-GTA-1-7 and tRNA:Tyr-GTA-1-8) and at least 700 nucleotides upstream and 300 nucleotides downstream sequences. Bacterial artificial chromosome (BAC) clone CH322-135M19 containing the three tRNA^Tyr-GTA^ genes was used as a template for PCR amplification of the genomic fragment, and pEntr (Thermo Scientific) was used as a template for PCR amplification of a plasmid backbone. Phusion high-fidelity DNA polymerase (New England Biolabs) was used for PCR, in combination with the primer pairs listed in Supplementary Table 1. The NEBuilder online tool (New England Biolabs) was used to design the primers. The resulting construct was sequence verified and cloned into the plasmid pBID^24^ using the restriction enzymes KpnI and XbaI. This plasmid was used for embryo injection following standard procedures, using VK22 and VK20 genomic landing sites for transgene insertion on the second and third chromosome, respectively.

Meiotic recombination was used to generate recombinant chromosomes that contain both the UAS-TyrRS and a tRNA^Tyr^ transgene on either the second or the third chromosome. Subsequently, stocks were generated that contain UAS-TyrRS, tRNA^Tyr^ transgenes on both the second and third chromosome. These stocks were crossed to the relevant GAL4 driver to obtain the experimental genotypes. For controls overexpressing the tRNA^Tyr^ transgenes alone, stocks were generated that contain tRNA^Tyr^ transgenes on both the second and third chromosome. The same approach was used to generate stocks that contain UAS-GlyRS and tRNA^Tyr^ transgenes on the second and third chromosome.

### Northern blotting

To evaluate tRNA levels in *Drosophila* third instar larvae, HEK 293T cells, or mouse spinal cord, total RNA was extracted using the TRI reagent (Zymo Research). For tRNA northern analysis, total RNA was loaded on denaturing 15% polyacrylamide gels and blotted onto Hybond-N+ membranes (GE Healthcare Life Sciences). The membrane was hybridized with 5′ end-labelled DNA oligo probes (Supplementary Table 2) at 42°C in a hybridization buffer containing 6 × saline-sodium citrate (SSC), 0.01 M sodium phosphate pH 6.8, 1 mM EDTA, 0.25% sodium dodecyl sulfate (SDS) and 100 μg/ml salmon sperm DNA. Blots were washed with 1× SSC, 0.1% SDS at 42°C (2 times 15 min). Phosphor screens were exposed to the blots for at least 2 h and imaged on a Typhoon FLA7000 scanner (GE Healthcare). Band intensity was determined using Image lab 6.1 (Bio-Rad Laboratories). After detection of the tRNA^Tyr^ bands, the membrane was stripped by addition of 100 ml boiling 0.1× SSC, 0.1% SDS solution. After 15 min, this procedure was repeated twice. Next, the membrane was re-probed with a methionyl-tRNA (tRNA^Met^) or valyl-tRNA (tRNA^Val^) probe (Supplementary Table 2) and imaged as described above. The tRNA^Met^ or tRNA^Val^ band intensities were used to normalize the corresponding tRNA^Tyr^ band intensities, thus serving as a loading control.

### Adult offspring frequency

To evaluate adult offspring frequency, virgin females heterozygous for *actin5C-GAL4* driver over a balancer chromosome were crossed to males homozygous for UAS-TyrRS transgenes alone or combined with tRNA^Tyr^ transgenes, and maintained at 25°C. F1 progeny with and without balancer were counted for 9 days after eclosion of the first F1 flies, and the percentage of non-balancer flies was calculated and plotted as a percentage of the driver-only control.

### Analysis of sensory neuron dendritic morphology

Class IV multidendritic (MD) sensory neuron morphology imaging was performed on living third instar larvae. Methodological details can be found in the Supplementary material available at *Brain* online.

### Automated negative geotaxis assay to analyse motor performance

To assay motor performance, an automated rapid iterative negative geotaxis assay (RING-assay) was used, which was previously established in the Storkebaum lab.^10^ Methodological details can be found in the Supplementary material available at *Brain* online.

### Analysis of larval muscle innervation

To analyse innervation of larval muscle 24 and synapse length on muscle 8, third instar larvae were dissected in hemolymph-like 3 (HL3) buffer and fixed in Bouin’s for 3 min. After permeabilization and blocking (10% goat serum), immunostaining was performed with anti-Discs large 1 [anti-Dlg1; developmental studies hybridoma bank (DSHB), 1/200]. Images were made of muscle eight in abdominal segment five using a Leica SP8 laser scanning confocal microscope with 20× Plan-Apochromat objective (0.8 numerical aperture). Maximum intensity projections of z-stacks comprising the entire neuromuscular junction (NMJ) were used to measure the synapse length. The innervation status of muscle 24 in abdominal segment 6 was evaluated by scoring the presence (‘innervated’) or absence (‘denervated’) of Dlg1 staining.

### Analysis of protein synthesis by *in vivo* cell-type-specific fluorescent non-canonical amino acid tagging

For fluorescent non-canonical amino acid tagging (FUNCAT) analysis of protein synthesis,^10,25,26^ 4 h egg collections were performed and animals were raised on Jazz-Mix *Drosophila* medium (Fisher Scientific) at 25°C; 72 h after egg laying (AEL), larvae were transferred to 4 mM azidonorleucine (ANL)-containing medium for 48 h. The larval central nervous system (CNS) was dissected in ice-cold HL3 solution and fixed in 4% paraformaldehyde (PFA) for 30 min at RT. After fixation, the CNSs were washed 3 × 10 min with PBST (1× PBS pH 7.2 containing 0.2% Triton X-100) and 3 × 10 min with PBS, pH 7.8. Metabolically labelled proteins were tagged by ‘click chemistry’ [Copper-Catalysed (3 + 2)-Azide-Alkyne-Cycloaddition Chemistry (CuAAC)] using the fluorescent tag 5-carboxytetramethylrhodamine (TAMRA). The FUNCAT reaction mix was assembled in a defined sequence of steps. Triazole ligand (1:1000), TAMRA-alkyne tag (1:5000), tris(2-carboxyethyl)phosphine (TCEP) solution (1:1000) and CuSO_4_ solution (1:1000) were added to PBS pH 7.8. After each addition, the solution was mixed thoroughly for 10 s and at the end for 30 s using a high-speed vortex. Larval brains were incubated with 200 μl of FUNCAT reaction mix overnight at 4°C on a rotating platform. The next day, the brains were washed three times with PBS-Tween and three times with PBST for 10 min. Finally, larval CNSs were mounted in VectaShield mounting medium (Biozol, VEC-H-1000-CE) and stored at 4°C until imaging using a Leica SP8 laser scanning confocal microscope. For image acquisition, identical confocal settings were used for all samples of a given experiment. Fluorescence intensities were quantified using ImageJ/FIJI software (National Institutes of Health). The mean intensity of all cells within one motor neuron cluster from each ventral nerve cord were used as single data-points for statistical analysis.

### *In vivo* quantification of tRNA^Tyr^ bound to TyrRS

Whole larvae ubiquitously (*actin5C-GAL4*) expressing WT TyrRS, TyrRS-G41R, TyrRS-153-156delVKQV or TyrRS-E196K were snap frozen in liquid nitrogen and stored at −80°C. Levels of tRNA^Tyr^ associated with TyrRS variants were quantified as described in a previously published detailed protocol.^27^ Each independent experiment was carried out with eight snap-frozen larvae. Briefly, frozen larvae were ground up, subjected to UV-crosslinking at 254 nm, 400 mJ/cm^2^ (UVP-TL-2000 Translinker), after which lysis buffer [20 mM Tris-HCl pH 7.4, 15 mM NaCl, 1% nonidet P-40 (NP-40), 0.1% Triton-100, 0.5% SDS, 2 mM dithiothreitol (DTT), protease inhibitor] was added. Protein-G-Dynabeads (Invitrogen) were coupled with two anti-TyrRS antibodies [2 µg each: mouse monoclonal (Abcam, ab50961) and rabbit polyclonal (Bethyl Labs, A305-064A)], and incubated with the lysed tissue overnight at 4°C. Beads were precipitated by centrifugation and washed with buffer [20 mM Tris-HCl pH 7.4, 100 mM NaCl, 1% NP-40, 0.1% Triton-100, 0.5% sodium deoxycholate (SDC), 2 mM DTT]. The tRNA bound to TyrRS (co-immunoprecipitated with the TyrRS-antibody-coated Dynabeads) was extracted with phenol:chloroform (Sigma) and subjected to quantification. By this extraction, the tRNA is completely deacylated. For quantification of the TyrRS-bound tRNA, we used a Cy3-labelled fluorescent stem-loop RNA/DNA oligonucleotide^28^ that ligates to the free unpaired 3′-NCCA end of tRNA^Tyr^ (5′-pCGCACUGCdTdTXdTdTdGdCdAdGdTdGdCdGdTdGdGdN-3′). The labelled tRNA^Tyr^ was loaded on a 10% denaturing polyacrylamide gel and the fluorescent signals were quantified using ImageJ.

For the TyrRS protein quantification by the capillary electrophoresis immunoblotting system (Jess, ProteinSimple), mouse monoclonal antibody (Abcam, ab50961); 1:25 dilution, was used. Whereas capillary electrophoresis allows precise quantification of protein levels, fluorescent detection of tRNA^Tyr^ is semi-quantitative owing to the variable background signal, even within a single gel. Therefore, measurements of tRNA^Tyr^ bound to TyrRS variants should be regarded as estimates and are not suitable for statistical analysis.

### Evaluation of protein synthesis in adult fly heads using threonine-derived non-canonical amino acid tagging

Flies carrying a conditional ubiquitous driver (*tub Gal80^ts^; tub-GAL4*) were crossed to either control (*w^1118^*), UAS-TyrRS-G41R, UAS-TyrRS-153-156delVKQV or UAS-TyrRS-E196K flies. To avoid developmental lethality of the progeny, vials were kept at the permissive temperature (18°C) during development. After eclosion, adult flies were transferred to the restrictive temperature (29°C), allowing for the expression of the UAS-TyrRS transgenes. Three days later, flies were transferred to Jazz-Mix *Drosophila* medium containing 4 mM of the threonine analogue β-ethynylserine (βES). After 48 h on βES-containing medium, whole flies were snap frozen in liquid nitrogen and stored at −80°C until further use. Heads of frozen flies (20 to 25 heads per replicate) were collected using stainless steel sieves (Cole-Parmer; 710 μm pore size upper sieve; 425 μm pore size middle sieve, and bottom container; head fraction retained in the middle sieve) cooled with liquid nitrogen. Frozen heads were homogenized in lysis buffer [4-(2-hydroxyethyl)-1-piperazineethanesulfonic acid (HEPES) 50 mM, pH 7.4, 150 mM NaCl, 0.5% NP-40, 1% sodium deoxycholate, 1% SDS, 0.5% Triton-X-100, 5% glycerol and 1× EDTA-free protease inhibitor cocktail] using micropestles (Eppendorf) and incubated for 10 min on ice. The lysate was cleared by centrifugation (13 000 *g*, 30 min) at 4°C, after which the supernatant was transferred to a new tube, and the protein concentration was determined by Pierce™ BCA Protein Assay (Thermo Fisher Scientific); 100 μg of protein lysate was transferred to a new tube and diluted to 90 μl total volume using elution buffer (50 mM HEPES, pH 7.4, 150 mM NaCl, 1% Triton-X-100, 1 × EDTA-free protease inhibitor cocktail). Then 10 μl of a 10× concentrated CuAAC reagent mixture, consisting of 10 mM CuSO_4_·5 H_2_O, 20 mM Tris-hydroxypropyltriazolylmethylamine (THPTA), 10 μM Sulfo-Cy5-azide (Jena Bioscience) and 100 mM sodium ascorbate, was added, and the reaction mixture was agitated at 37°C in an orbital shaker at 550 rpm for 30 min. Next, the protein lysate was precipitated by the addition of 400 μl of ice-cold acetone, and the protein pellet was collected by centrifugation (5000*g*, 20 min) at 4°C. Protein pellets were allowed to air dry and were dissolved in 1× Laemmli sample buffer (400 mM DTT, 200 mM Tris-HCl, pH 6.8, 8% SDS, 40% glycerol, bromophenol blue, 3 M urea and 5% β-mercaptoethanol). Samples were heated at 70°C for 10 min and 20 μg of protein for each sample was loaded onto a 1.5 mm 12% polyacrylamide gel (Bio-Rad). Gels were run at 80 V for the stacking gel and 120 V for the running gel. After the run, gels were stained overnight with the fluorescent protein gel stain Sypro Ruby (Thermo Fisher Scientific). In-gel fluorescence was measured using a Typhoon™ 5 gel scanner (Cytiva) using the 635 nm laser and the 670BP30 filter for the Cy5 channel (newly synthetized proteins) and the 488 nm laser and the 655–685 filter for the Sypro channel (total protein amounts).

### Ribosome profiling

Ribosome profiling was carried out as described previously.^11^ Between 25 and 50 flash frozen *Drosophila* heads per sample were lysed by grinding in polysome lysis buffer [10 mM Tris-HCl, pH 7.4, 5 mM MgCl_2_, 100 mM KCl, 1% Triton X-100, 2% sodium deoxycholate, 2 mM DTT and 100 µg/ml cycloheximide (CHX)], followed by gentle shearing with a 26-guage needle. The lysate was cleared by centrifugation at 14 000*g* for 10 min at 4°C. The supernatant was digested with 1.5 µl RNaseI (Biosearch technologies, N6901K) in a final volume of 200 µl for 15 min at 22°C, by gentle shaking at 300 rpm. The digestion was stopped with 1.5 µl SUPERase-In RNase Inhibitor (Invitrogen, A42694). Ribosome-protected fragments (RPFs) were purified using the Zymo Quick RNA Microprep kit (R1050). RNA size selection was performed by denaturing gel electrophoresis. RNA fragments in the range of 20 to 40 nt were purified from the gel using the Immagina polyacrylamide gel electrophoresis (PAGE) Extraction Kit PAGExt (KGE00-12). Ribosomal RNA was depleted using the NEBNext rRNA Depletion Kit v2 (human/mouse/rat, E7400S). Sequencing libraries were prepared by direct ligation of the adapters, using the QIAseq microRNA (miRNA) Library kit (Qiagen, 331502) in combination with the QIAseq miRNA 96 Index kit IL UDI-A (331905). The library size distribution was analysed on an Agilent 2100 Bioanalyzer DNA Chip (Agilent Technologies), and sequencing was performed with the Illumina NovaSeq 6000 Sequencing System.

Ribosome profiling was performed on two independent biological replicates. Sequenced reads were depleted from adapter sequences and uniquely mapped to the *Drosophila melanogaster* genome (BDGP6.32). After mapping, the reproducibility of the libraries was assessed by Pearson correlation and because of their very high reproducibility (i.e. for TyrRS-153-156delVKQV—0.89; for TyrRS-E196K—0.91; for driver-only—0.94), the reads from two independent replicates were pooled and used as a single mega set for the subsequent analysis. RPFs were calibrated to determine the A-site position of each codon following the calibration tool (https://github.com/AlexanderBartholomaeus/MiMB_ribosome_profiling).^29^ Concisely, RPFs were binned into equal read lengths and plotted individually to the vicinity of the start codon to determine the offset to the P site at the start codon. The A site was determined by adding three nucleotides to the position of the respective P site. The A-site ribosome dwelling occupancies (frequencies) were calculated by summing them up for each transcript and normalized on the transcript background, i.e. by the mean of randomized reads to consider differences in codon frequencies across transcripts.^30^ To compare the differences in ribosome dwelling occupancy (frequency) at the A-site codon between different samples, the summed-up A-site codon occupancies across all transcripts for each sample were then divided and presented as differential A-site codon occupancies.

### Validation of tRNA^Tyr^ expression constructs in HEK 293T cells

HEK 293T cells were cultured in Dulbecco's Modified Eagle's Medium (DMEM) (Gibco, 41966-029) supplemented with 10% fetal bovine serum (FBS) (Gibco, A5209401) and 1% penicillin-streptomycin (Gibco, 15070063). For transfection, cells were seeded in 12-well plates and grown to approximately 70% confluency under the same conditions. Before transfection, cells were washed with PBS and incubated in optimized minimal essential medium (Opti-MEM) (Gibco, 31985-062).

Cells were transfected with equimolar amounts of the following plasmids: cytomegalovirus promoter-green fluorescent protein (CMV-GFP), U6-tRNA^Tyr^ no intron, U6-tRNA^Tyr^ with intron or U6-tRNA^Gly^ (please refer to the ‘AAV vector production’ section for a more detailed description of these constructs). Lipofectamine 3000 (Invitrogen, L3000-015) was used according to the manufacturer’s instructions. After 4 h of incubation with the Lipofectamine-plasmid mixture, the medium was replaced with DMEM; 24 h post-transfection, cells were harvested and the pellets snap-frozen for northern blot analysis.

### Mouse genetics

The mouse model of dominant intermediate CMT-C carrying the patient-associated E196K mutation knocked into *Yars1* was previously described.^13,31^ The official allele designation is *Yars1*^tm1.1Rwb^, MGI:725843. All animal studies were performed in compliance with the Guide on the Care and Use of Laboratory Animals and procedures were reviewed and approved by the Animal Care and Use Committee of The Jackson Laboratory (ASU01026) and the Institutional Biosafety Committee (2019001). Mice were housed in pressurized, individually ventilated cages with food and water provided *ad libitum* and a 14:10 h light:dark cycle.

### AAV vector production

Four AAV9 vectors were produced for use in this study. First, the mature sequence of tRNA^Tyr-GUA^ (GUA anticodon, human tRNA-Tyr-GTA-1-1) was driven by the mouse U6 promoter. This construct is referred to as ‘U6-tRNA^Tyr^ no intron’. Despite two codons for tyrosine (UAU and UAC), the mouse and human genomes only encode one tRNA^Tyr^ isoacceptor, with GUA anticodon, which decodes both codons through G-U base pairing at the wobble position. The mature tRNA^Tyr-GUA^ sequence is identical between mouse and human. Second, the same tRNA^Tyr^ gene, but containing its endogenous intron, was driven by the mouse U6 promoter. This construct is referred to as ‘U6-tRNA^Tyr^ with intron’. Third, tRNA^Gly-GCC^ (GCC anticodon, gene *n-TGgcc5*, tRNA-Gly-GCC-2-1) was driven by the mouse U6 promoter as a control to confirm the specificity of phenotypic rescue by tRNA^Tyr^ overexpression. This construct is referred to as ‘U6-tRNA^Gly^’. Fourth, the human TyrRS coding region was driven by a CMV promoter. The construct also included a C-terminal V5 epitope tag and SV40 polyA. This construct is referred to as ‘CMV-TyrRS’. All constructs were packaged by Andelyn (Columbus) into AAV9 serotype capsids using a three-plasmid packaging protocol. Titres were determined by a digital droplet PCR assay of DNase resistant particles.

### *In vivo* AAV delivery

AAV9 vectors were administered using intracerebroventricular (ICV) injection into the lateral ventricle of neonatal mice (postnatal Days 0–2). For consistency, all vectors were delivered at 1.75 × 10^11^ viral genomes per mouse. Titres and volumes used were: 4.6 × 10^13^/ml, 3.8 µl for U6-tRNA^Tyr^ no intron, 5.1 × 10^13^/ml, 3.4 µl for U6-tRNA^Tyr^ with intron, 3.5 × 10^13^/ml, 5 µl for U6-tRNA^Gly^ and 5.4 × 10^13^/ml, 3.2 µl for CMV-TyrRS. The pups were anaesthetized by hypothermia, placing them on ice briefly until they were no longer moving. The virus was delivered into the lateral ventricle using a Hamilton syringe with a Neuros adapter to achieve a fixed 2 mm of penetration. Given the translucent skin and skull at this age, this was done by hand without stereotaxis. This procedure takes under 1 min and the pups recovered from hypothermia on a controlled warming pad (35˚C) until they were active.

### Inverted grid test

Motor performance was assessed using the wire hang test of grip strength and endurance, also known as the inverted grid test.^32^ In this test, mice are placed on a wire grid, which is then inverted ∼30 cm above a pen with bedding. This is repeated for three 1-min trials, with 60 s intervals between trials. The average time-to-fall across the three trials is reported.

### Neurophysiology

An AD Instruments PowerLab system was used to determine nerve conduction velocity. Mice were anaesthetized with 2% isoflurane and placed on a heating pad to maintain body temperature. To perform sciatic motor nerve conduction velocity (NCV) measurements, the compound muscle action potential (CMAP) amplitude was recorded from the muscles of the right hind paw following stimulation at the ankle and the hip, near the sciatic notch. The distance between the electrodes was measured and the differences in latency from stimulation to electromyography signal (EMG) was determined. NCV was calculated using the following equation: distance/(hip latency–ankle latency) and reported in m/s.

### Nerve histology

The motor branch of the femoral nerve was fixed in place using a drop of 2% paraformaldehyde and 2% glutaraldehyde in a 0.1 M cacodylate buffer. The nerves were then dissected free, post-fixed overnight in the same solution and rinsed in PBS. Samples were dehydrated and plastic embedded for sectioning.^32^ Semi-thin sections (1 μm) were placed on slides and stained with toluidine blue for light microscopy. Images were collected on a Nikon Eclipse 600 microscope with a 40× objective. Axon cross-sectional area was quantified using ImageJ, and outputs were manually confirmed by visual inspection and compared between genotypes. Images were montaged using Adobe’s Photoshop photomerge tool if they did not fit in one 40× field of view. Images were then opened in Fiji/ImageJ and converted to greyscale, flattened and converted on an 8-bit image. The scale was set based on the pixel size of the camera. Using the threshold function, thresholds were set such that only myelin around the axons was visible, and using the analyse particles function, where size = 1–Infinity and Circularity = 0.5–1.0, the program generated regions of interest (ROI). These ROIs are then manually assessed and curated to ensure all the selected areas are within the axon and myelin, and no axons were missed. Any axons initially missed were added manually using the wand tool with the tolerance set to 30 in legacy mode. Similarly, anything that was not an axon that was selected as an ROI was removed. The data produced from this included the area of each axon, allowing to report the size distribution of axons within the nerves. The distribution of axon areas (*x*-axis) is presented as a cumulative histogram, with the per cent of the sample population on the *y*-axis.

### Western blotting

The spinal cord of wild type mice with or without ICV treatment with AAV9-CMV-TyrRS were collected at 4 weeks of age. Tissue was homogenized in radioimmunoprecipitation assay (RIPA) buffer (150 mM NaCl, 1% NP-40, 0.5% sodium deoxycholate, 0.1% SDS and 50 mM Tris, pH 8.0) using a glass/glass dounce homogenizer. Total protein concentration of the homogenates was determined using a BCA assay (Pierce #23227), and 30 µg of boiled protein lysate was loaded onto a 4%–20% gradient gel (Bio-Rad, 4561093) for SDS-PAGE. Protein was then transferred to a polyvinylidene fluoride (PVDF) membrane by immersion blotting in transfer buffer (25 mM Tris and 192 mM glycine, pH 8.3, plus 10% MeOH). The blot was blocked in a 5% solution of powdered milk in Tris-buffered saline plus 0.1% Tween-20 and then probed with a primary antibody against the V5-epitope tag (ThermoFisher, R961-25, 1:2500), and a secondary horseradish peroxidase (HRP)-conjugated anti-mouse antibody (Jackson ImmunoResearch, 615-035-214, 1:1000). Antibodies were diluted in blocking solution. Signal was visualized using enhanced chemiluminesence (ThermoFisher, 34577 or 32209, depending on signal strength) and exposed to autoradiography film. Blots were then stripped by incubating with agitation in a solution of 62.5 mM Tris, pH 6.8, 2% SDS and 0.8% β-mercaptoethanol at 50°C for 30 min and re-probed with an antibody against β-Actin (Sigma, A2228, 1:2000) as a loading control using the same conditions.

### Quantitative real-time PCR

To assess expression of activating transcription factor 4 (ATF4) target genes as a readout of activation of the integrated stress response, total RNA was prepared from snap-frozen spinal cords from mice at 6 months of age. Reverse transcription was performed on 1 µg of RNA using reverse transcriptase (Invitrogen, 18080400) according to the manufacturer’s protocol. ATF4-target genes known to be induced in *Gars1* and *Yars1* mouse models of CMT disease were assayed using β-actin as an internal standard. Target genes and primer combinations are listed in Supplementary Table 3. Samples were run on a QuantStudio 7 real-time PCR machine with SYBR green reagents (ThermoFisher, A46109). Changes in expression were determined using the ΔΔC_t_ method.

### Statistics

All data are presented as mean ± standard error of the mean (SEM) and differences were considered significant when *P* < 0.05. Animals or samples were assigned to the various experimental groups based on their genotype. For a given experiment, samples from the different experimental groups were processed in parallel and analysed in random order.

Statistical tests were performed using GraphPad Prism software. Before analysis, a Robust regression and Outlier removal method (ROUT) was performed to detect statistical outliers. This non-linear regression method fits a curve that is not influenced by outliers. Residuals of this fit were then analysed by a test adapted from the false discovery rate approach, to identify any outliers. All data-points that were considered outliers were excluded from further data analysis. Normality and homoscedasticity of all data sets was assessed by Shapiro–Wilk and Brown–Forsythe (F-test for *t*-tests) tests, respectively. Subsequent statistical tests were only performed if all assumptions were met.

For comparison of normally distributed data of more than two groups, one-way ANOVA followed by Tukey’s or Sidak’s multiple comparisons *post hoc* test was used, provided that the standard deviations were not significantly different between groups (equal variance, evaluated by Brown–Forsythe test and Bartlett’s test). For comparison of normally distributed data of more than two groups with unequal variance, Brown–Forsythe and Welch ANOVA followed by Dunnett’s T3 multiple comparisons *post hoc* test was used. For comparison of not normally distributed data, the Kruskal–Wallis test with Dunn’s multiple comparison test was used. Fisher’s exact test with Bonferroni correction was used to analyse adult offspring frequency and innervation status of larval muscle 24. The Kolmogorov–Smirnov test was used to compare the cumulative frequency of axon diameters between genotype groups.

## Results

### tRNA^Tyr^ overexpression rescues peripheral neuropathy and mRNA translation in CMT-*YARS1 Drosophila* models

The *Drosophila* genome contains 10 tRNA^Tyr^ genes, all with GUA anticodon to decode both Tyr codons, UAU and UAC. We assembled three copies of a genomic DNA fragment that contains a cluster of three tRNA^Tyr^ genes (tRNA-Tyr-GTA-1-6, tRNA-Tyr-GTA-1-7, tRNA-Tyr-GTA-1-8, nomenclature based on the genomic tRNA database http://gtrnadb.ucsc.edu/) in a transgenic construct (Fig. 1A). This transgene was site-specifically inserted into genomic landing sites on the second and third chromosomes. In *Drosophila* larvae carrying two or four copies of this transgene (corresponding to 18 or 36 extra tRNA^Tyr^ gene copies), tRNA^Tyr^ expression levels were increased by ∼71% and ∼100%, respectively, as revealed by northern blotting (Fig. 1B and C).

**Figure 1:**
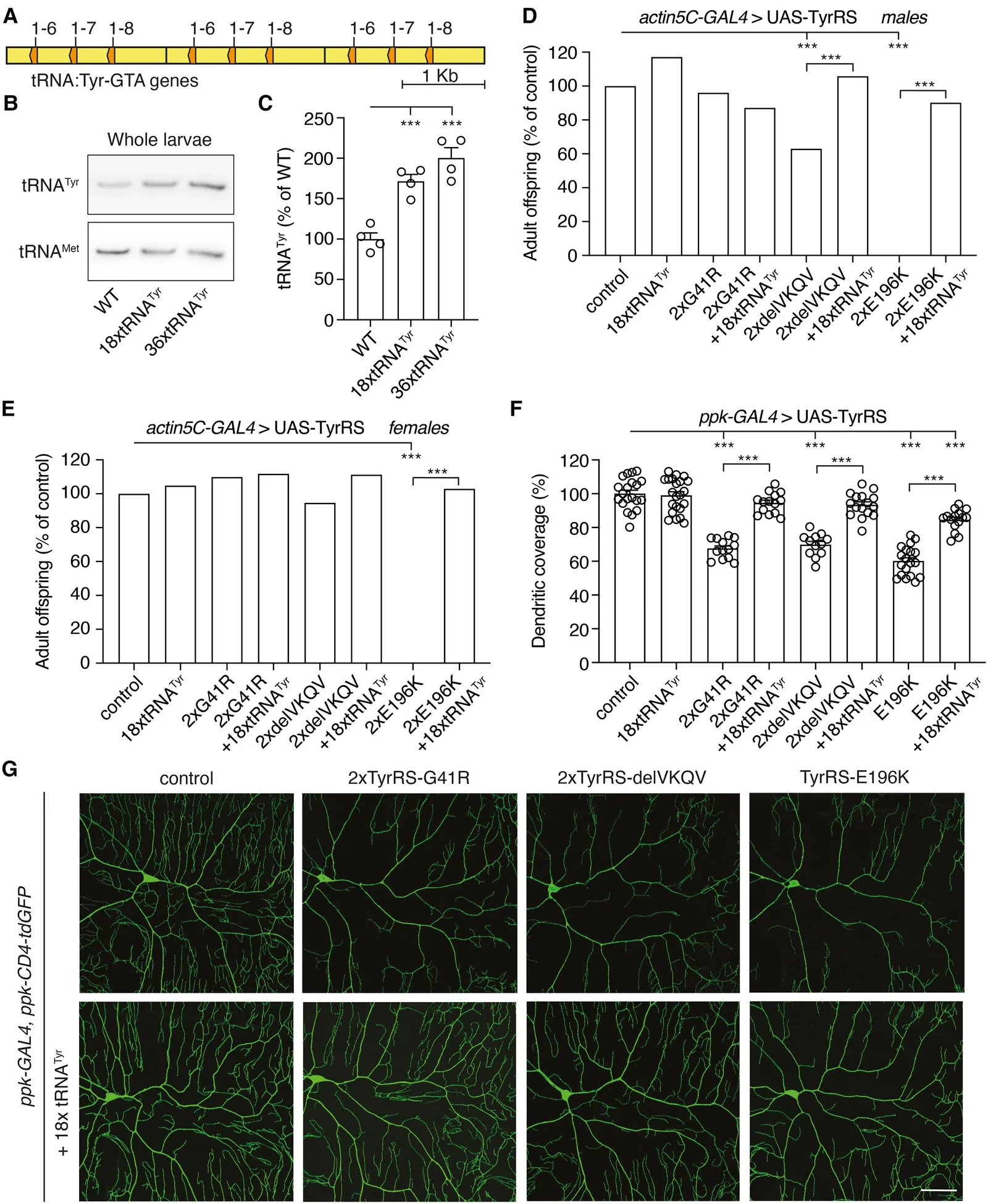
**tRNA^Tyr^ overexpression rescues peripheral neuropathy phenotypes in *Drosophila* CMT-*YARS1* models**. (**A**) Schematic overview of the 9xtRNA^Tyr-GTA^ transgene used for tRNA^Tyr-GUA^ overexpression in *Drosophila*. (**B**) Representative northern blot to evaluate tRNA^Tyr^ levels in RNA extracts from third instar whole larvae. Transgenic larvae containing 18 (18xtRNA^Tyr^) or 36 (36xtRNA^Tyr^) extra tRNA^Tyr^ gene copies were compared with wild-type (WT) larvae. tRNA^Met^ was used as loading control, obtained after stripping and re-probing the blot with a tRNA^Met^ probe. (**C**) Quantification of tRNA^Tyr^ levels in WT, 18xtRNA^Tyr^ and 36xtRNA^Tyr^ larvae. Data are shown as % of WT. *n* = 4 independent samples per genotype; ***P < 0.005 by one-way ANOVA with Tukey’s multiple comparisons test. (**D** and **E**) Adult offspring frequency (% of driver-only control) of male (**D**) and female (**E**) flies ubiquitously (*actin5C-GAL4*) expressing two copies of G41R, 153-156delVKQV or E196K TyrRS transgenes, in the presence or absence of 18xtRNA^Tyr^ transgenes. *n* > 92 F1 flies per cross; ***P < 0.0001 by Fisher’s exact test with Bonferroni correction. (**F** and **G**) Quantification (**F**) and representative images (**G**) of dendritic coverage (% of driver-only control) of class IV multidendritic sensory neurons in the larval body wall upon selective expression of TyrRS transgenes in these neurons (*ppk-GAL4*), in the presence or absence of 18xtRNA^Tyr^. *n* = 12–22 animals per genotype; ***P < 0.0001 by Brown–Forsythe and Welch ANOVA with Dunnett’s T3 multiple comparisons test. Scale bar = 50 μm. TyrRS=tyrosyl-tRNA synthetase.

Next, we evaluated the effect of tRNA^Tyr^ overexpression on peripheral neuropathy phenotypes and defective protein synthesis in three *Drosophila* CMT-*YARS1* models, expressing human TyrRS-G41R, TyrRS-153-156delVKQV or TyrRS-E196K variants.^10^ Ubiquitous expression (*actin5C-GAL4*) of CMT-TyrRS variants induced developmental lethality to various extents: TyrRS-E196K induced developmental lethality in both male and female offspring, expression of TyrRS-153-156delVKQV significantly reduced adult male offspring frequency, whereas TyrRS-G41R did not induce developmental lethality (Fig. 1D and E). Strikingly, tRNA^Tyr^ overexpression fully rescued the developmental lethality induced by TyrRS-E196K and TyrRS-153-156delVKQV expression (Fig. 1D and E). For all three CMT-TyrRS variants, selective expression in class IV multidendritic sensory neurons (*ppk-GAL4*) in the larval body wall resulted in reduced dendritic coverage (Fig. 1F and G). Transgenic tRNA^Tyr^ overexpression rescued this sensory neuron morphology defect (Fig. 1F and G). Pan-neuronal expression of TyrRS-E196K, but not TyrRS-G41R or TyrRS-153-156delVKQV, resulted in reduced climbing speed of adult flies as revealed by an automated negative geotaxis assay.^10^ This adult motor performance deficit was fully rescued by tRNA^Tyr^ overexpression (Fig. 2A). Furthermore, selective expression of TyrRS-E196K, but not TyrRS-G41R or TyrRS-153-156delVKQV, in motor neurons (*OK371-GAL4*) induced larval neuromuscular junction (NMJ) defects: larval muscle 24, a distal muscle innervated by segmental motor nerve a (SNa), was denervated in ∼44% of the cases (Fig. 2B) and the neuromuscular synapse length on larval muscle 8 was reduced by ∼45% (Fig. 2C and D). Again, these larval NMJ defects were rescued by tRNA^Tyr^ overexpression (Fig. 2B-D). Finally, *in vivo* cell-type-specific FUNCAT^10,25,26^ revealed that expression of any of the three CMT-TyrRS variants in motor neurons reduced *de novo* protein synthesis in the range of ∼49% to ∼66%. Overexpression of tRNA^Tyr^ rescued this protein synthesis defect for each of the three CMT-TyrRS variants (Fig. 2E and F). Together, these data provide genetic evidence that depletion of the cellular free tRNA^Tyr^ pool—presumably due to tRNA^Tyr^ sequestration by CMT-mutant TyrRS variants—triggers peripheral neuropathy and causes protein synthesis defects in *Drosophila* CMT-*YARS1* models.

**Figure 2:**
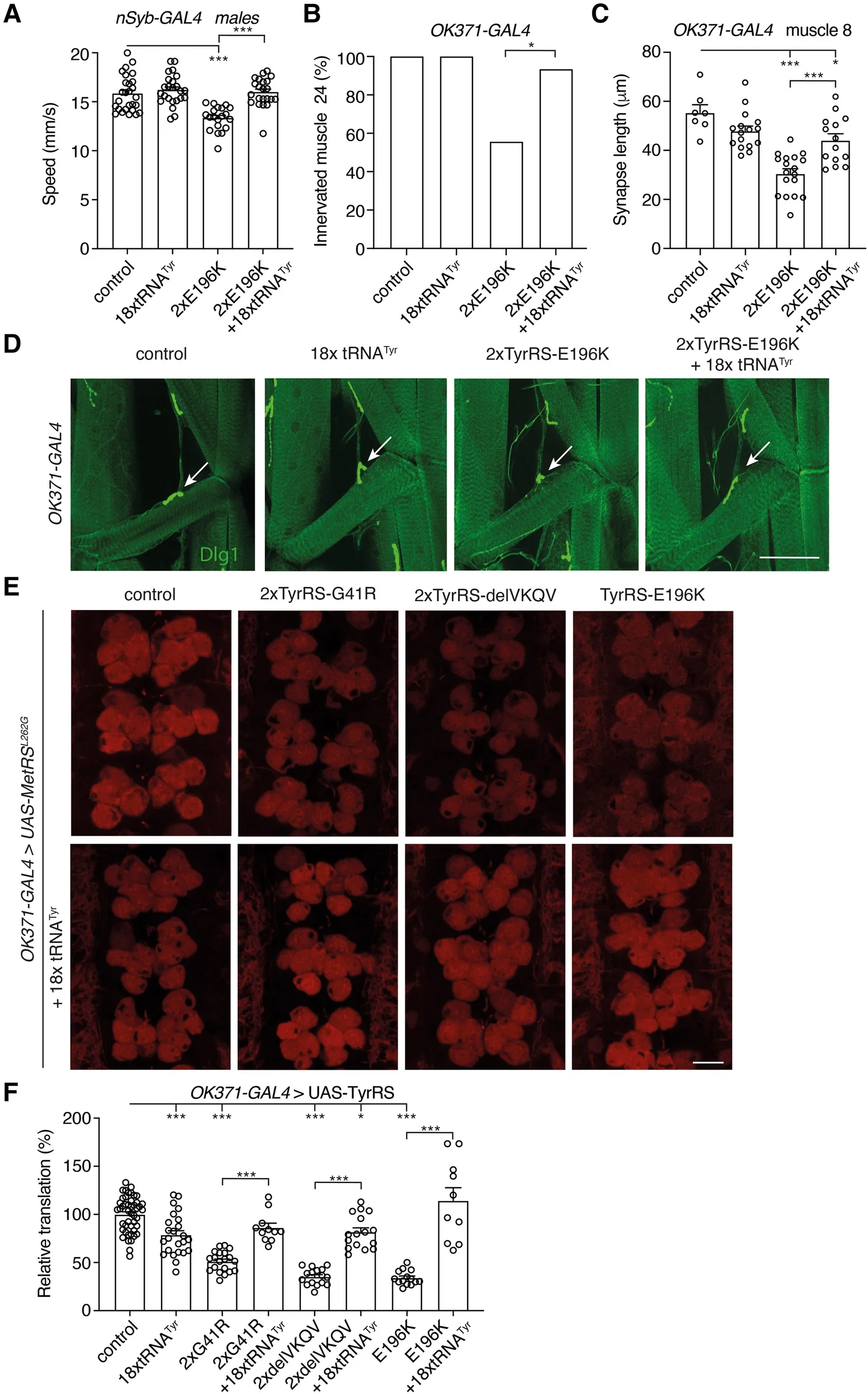
**tRNA^Tyr^ overexpression rescues peripheral neuropathy and inhibition of protein synthesis in *Drosophila* CMT-*YARS1* models.** (**A**) Climbing speed in an automated negative geotaxis assay of 7-day-old male flies with pan-neuronal (*nSyb-GAL4*) expression of TyrRS-E196K, in the presence or absence of 18xtRNA^Tyr^. *n* = 20–27 groups of 10 flies per genotype; ***P < 0.0001 by one-way ANOVA with Sidak’s multiple comparisons test. (**B**) Percentage of larvae with innervated muscle 24. TyrRS-E196K was selectively expressed in motor neurons (*OK371-GAL4*), in the presence or absence of 18xtRNA^Tyr^. *n* = 7–18 animals per genotype; *P < 0.05 by the Fisher’s exact test. (**C** and **D**) Quantification (**C**) and representative images (**D**) of synapse length on distal muscle 8 of larvae selectively expressing TyrRS-E196K in motor neurons (*OK371-GAL4*), in the presence or absence of 18xtRNA^Tyr^. *n* = 7–18 animals per genotype; *P < 0.05; ***P < 0.0005 by one-way ANOVA with Sidak’s multiple comparisons test. In **D**, neuromuscular junctions (NMJs) are visualized using Discs large 1 (Dlg1) immunostaining. The NMJ on muscle 8 is indicated with a white arrow, illustrating the reduced synapse length upon expression of TyrRS-E196K. This defect is rescued by tRNA^Tyr^ overexpression. Control is driver-only. Scale bar = 100 μm. Note that in the experiments shown in **A**–**D**, the G41R and 153-156delVKQV TyrRS mutants were not included, because these mutants did not display significant deficits in negative geotaxis climbing behaviour, innervation of muscle 24 or synapse length on muscle 8. (**E** and **F**) Representative images (**E**) and quantification (**F**) of relative translation rate as determined by FUNCAT in motor neurons (*OK371-GAL4*) of larvae expressing G41R, 153-156delVKQV or E196K TyrRS transgenes, in the presence or absence of 18xtRNA^Tyr^. *n* = 10–46 animals per genotype; *P < 0.05, ***P < 0.005 by Brown–Forsythe and Welch ANOVA with Dunnett’s T3 multiple comparisons test. Scale bar=10 μm. FUNCAT=fluorescent non-canonical amino acid tagging; TyrRS = tyrosyl-tRNA synthetase.

### tRNA^Tyr^ overexpression does not rescue CMT-*GARS1 Drosophila* models

Because tRNA^Tyr^ sequestration by CMT-mutant TyrRS is expected to specifically deplete the cellular tRNA^Tyr^ pool without affecting the levels of other tRNAs, we next evaluated whether tRNA^Tyr^ overexpression specifically rescues CMT-TyrRS phenotypes. We previously reported that transgenic overexpression of tRNA^Gly^ does not rescue peripheral neuropathy phenotypes in *Drosophila* CMT-TyrRS models.^11^ In turn, tRNA^Tyr^ overexpression did not rescue the adult motor performance deficit (Fig. 3A and B), larval NMJ morphology defects (Fig. 3C) or sensory neuron dendritic morphology defects (Fig. 3D and E) induced by expression of any of three CMT-GlyRS variants (E71G, G240R, G526R). Together, these data confirm the specificity of the rescue of CMT-*YARS1* peripheral neuropathy phenotypes by tRNA^Tyr^ overexpression. More generally, our data indicate that peripheral neuropathy induced by mutations in a given tRNA synthetase can only be rescued by overexpression of the cognate tRNA(s).

**Figure 3:**
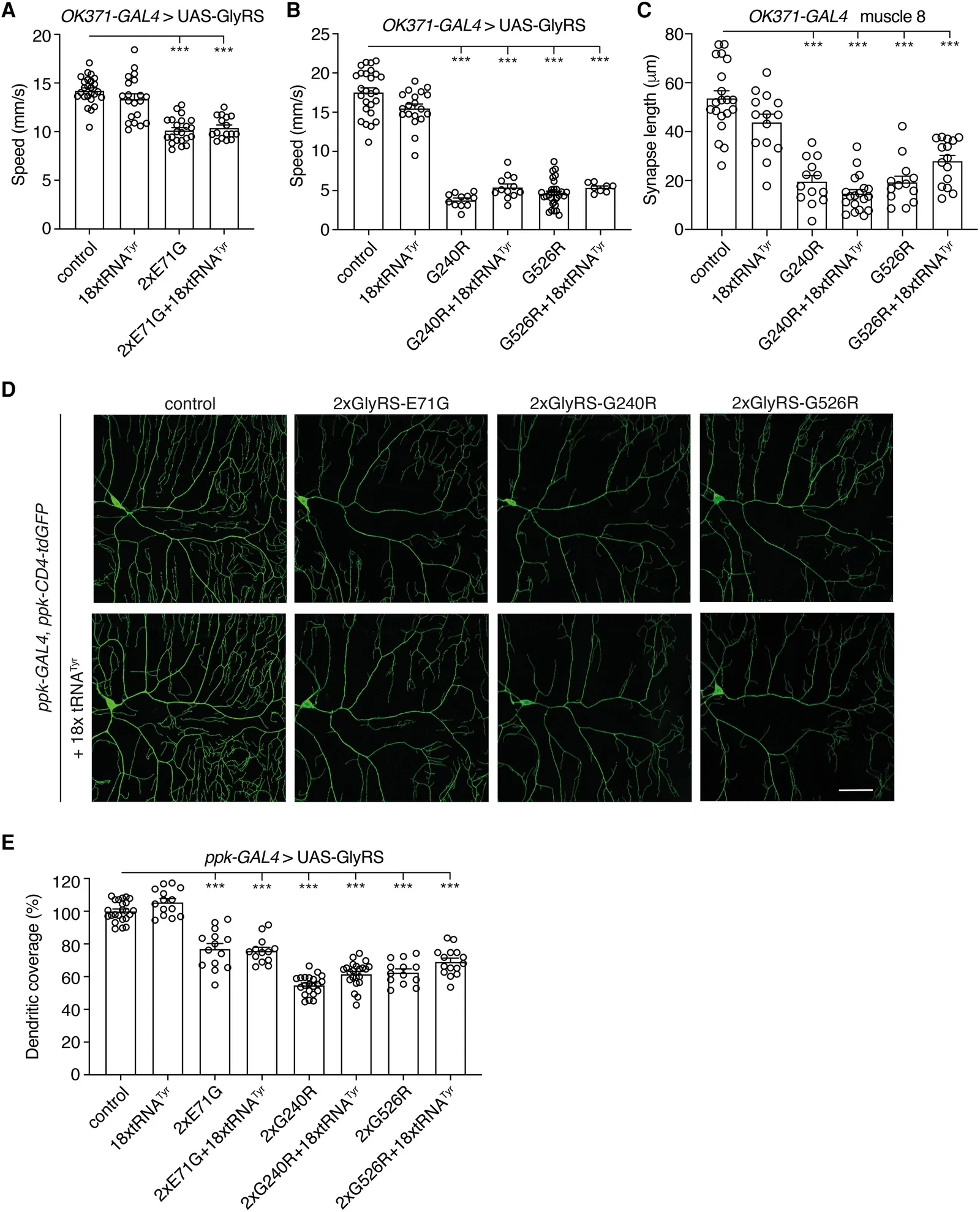
**tRNA^Tyr^ overexpression does not rescue peripheral neuropathy phenotypes in *Drosophila* CMT-*GARS1* models.** (**A** and **B**) Negative geotaxis climbing speed of 7-day-old male flies expressing E71G (**A**), G240R or G526R (**B**) GlyRS transgenes in motor neurons (*OK371-GAL4*), in the presence or absence of 18xtRNA^Tyr^. *n* = 8–30 groups of 10 flies per genotype; ***P < 0.005 by Brown–Forsythe and Welch ANOVA with Dunnett’s T3 multiple comparisons test (**A**) or Kruskal–Wallis test with Dunn’s multiple comparisons test (**B**). (**C**) Synapse length on distal muscle 8 of larvae selectively expressing GlyRS G240R or G526R in motor neurons, in the presence or absence of 18xtRNA^Tyr^. *n* = 13–20 animals per genotype; ***P < 0.0001 by one-way ANOVA with Sidak’s multiple comparisons test. (**D** and **E**) Representative images (**D**) and quantification (**E**) of dendritic coverage (% of driver-only control) of class IV multidendritic sensory neurons in the larval body wall upon selective expression of GlyRS transgenes in these neurons (*ppk-GAL4*), in the presence or absence of 18xtRNA^Tyr^. *n* = 13–23 animals per genotype; ***P < 0.0001 by one-way ANOVA with Sidak’s multiple comparisons test. Scale bar=50 μm. GlyRS=glycosyl-tRNA synthetase.

### CMT-TyrRS variants sequester tRNA^Tyr^ and trigger ribosome pausing at tyrosine codons

To provide *in vivo* biochemical evidence for tRNA^Tyr^ sequestration by CMT-TyrRS variants, we expressed WT human TyrRS or each of the three CMT-TyrRS variants ubiquitously in *Drosophila* larvae. Next, we immunoprecipitated human TyrRS variants from larval protein extracts and quantified the amount of bound tRNA^Tyr^ following our previously established protocol.^27^ The amount of tRNA^Tyr^ bound to each of the three CMT-TyrRS variants was markedly increased as compared with WT TyrRS (Fig. 4A). This indicates stronger association of tRNA^Tyr^ with CMT-TyrRS variants, consistent with tRNA^Tyr^ sequestration.

**Figure 4:**
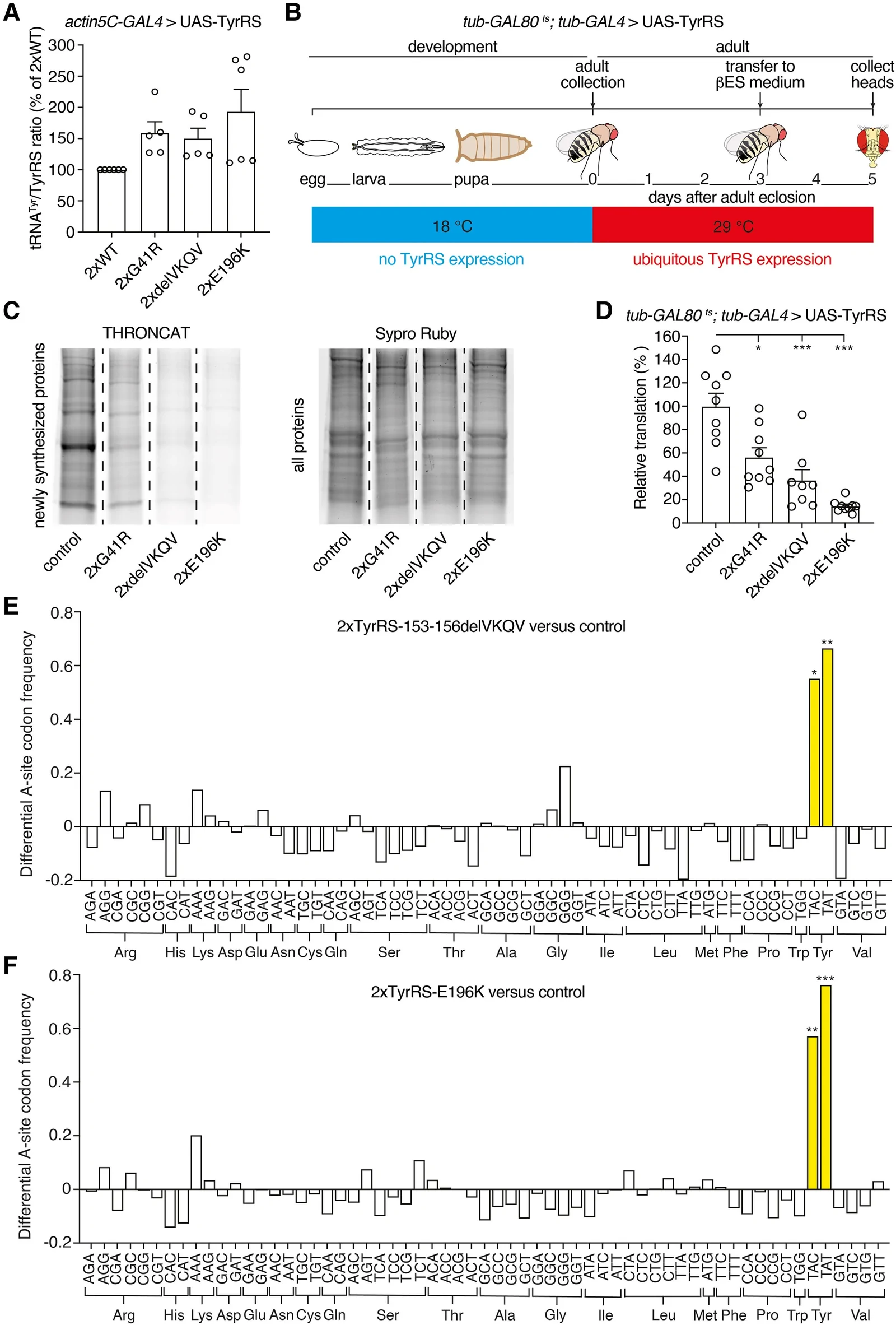
**tRNA^Tyr^ sequestration by CMT-mutant TyrRS leads to ribosome stalling at tyr codons**. (**A**) Measurement of tRNA^Tyr^ bound to TyrRS variants immunoprecipitated from lysates of whole third instar larvae that ubiquitously (*actin5C-GAL4*) express willd-type (WT) TyrRS, TyrRS-G41R, TyrRS-153-156delVKQV or TyrRS-E196K. tRNA^Tyr^/TyrRS ratio of WT TyrRS is set to 100%. *n* = 5–6 independent replicates. The variable distribution of data-points precluded statistical analysis. (**B**) Schematic overview of THRONCAT workflow. *Tub-GAL80^ts^; tub-GAL4* > UAS-TyrRS animals were raised at 18°C to suppress TyrRS transgene expression. After adult eclosion, flies were shifted to 29°C to induce ubiquitous TyrRS transgene expression. After 3 days, flies were transferred to β-ethynylserine (βES) containing medium for metabolic labelling, and 2 days later heads were collected and processed for THRONCAT. (**C**) *Left*: Representative image of Cyanine 5 (Cy5)-tagged newly synthesized proteins separated by SDS-PAGE gel electrophoresis and isolated from heads of flies ubiquitously expressing TyrRS-G41R, TyrRS-153-156delVKQV, TyrRS-E196K or no transgene (driver-only control). *Right*: Sypro Ruby was used to label all proteins as loading control. (**D**) Quantification of the intensity of newly synthesized proteins relative to total proteins, which is a proxy for global protein synthesis rate. The driver-only control is set to 100%. *n* = 8–9 samples per genotype, with each sample consisting of 20 to 25 fly heads; *P < 0.05, ***P < 0.005 by Brown–Forsythe and Welch ANOVA with Dunnett’s T3 multiple comparisons test. (**E** and **F**) Relative changes in the ribosome dwelling occupancy (frequency) at A-site codons in fly heads expressing TyrRS-153-156delVKQV (**E**) or TyrRS-E196K (**F**) compared with driver-only controls, as determined by ribosome profiling. The ribosome profiling data are pooled from two independent biological replicates. A common set of 1460 transcripts with a good coverage and expressed in all samples were used for the comparisons. *P < 0.05, **P < 0.01, ***P < 0.005 by paired-sample *t*-test for each codon; *P*-values were calculated using the t-distribution. TyrRS=tyrosyl-tRNA synthetase.

Next, we assessed whether this tRNA^Tyr^ sequestration would sufficiently deplete the cellular tRNA^Tyr^ pool to induce ribosome stalling at Tyr codons. To circumvent developmental lethality, we ubiquitously expressed the CMT-TyrRS variants from the adult stage onwards, using the GAL80 target system^23^ (Fig. 4B). To assess protein synthesis in adult flies, we exploited the recently described THRONCAT approach^33^ for metabolic labelling of newly synthesized proteins within a 48 h time frame, starting 3 days after the induction of TyrRS transgene expression. Next, protein extracts from fly heads were separated by SDS-PAGE, labelled proteins were detected by click chemistry and levels were compared with total protein. This experiment revealed strong inhibition of *de novo* protein synthesis by all CMT-TyrRS variants, i.e. by ∼44% for TyrRS-G41R, ∼63% for TyrRS-153-156delVKQV and ∼85% for TyrRS-E196K (Fig. 4C and D).

Next, ribosome profiling was performed on head extracts from flies ubiquitously expressing TyrRS-153-156delVKQV or TyrRS-E196K from the adult stage onwards, because these two TyrRS variants induced the most pronounced protein synthesis defect (Fig. 4C and D). This analysis revealed that Tyr codons were more frequently found vacant in the ribosomal A site when either of these two CMT-TyrRS variants were expressed (Fig. 4E and F). The ribosome dwell times at Tyr codons increased for both Tyr codons (TAC and TAT), for TyrRS-153-156delVKQV by ∼155% for TAC and ∼167% for TAT (Fig. 4E) and for TyrRS-E196K by ∼157% for TAC and ∼176% for TAT (Fig. 4F). Together, these data support the notion that shortage of tRNA^Tyr^ resulting from tRNA^Tyr^ sequestration by the CMT-mutant TyrRS variants increases ribosome dwelling at Tyr codons.

### AAV9-mediated tRNA^Tyr^ overexpression prevents peripheral neuropathy and ISR activation in *Yars1^E196K/E196K^* mice

To strengthen the potential relevance of our findings in *Drosophila* models for human CMT-*YARS1*, we turned to a knock-in CMT-*YARS1* mouse model, because this is the evolutionary closest available animal model to humans. *Yars1^E196K/E196K^* mice were previously reported to develop peripheral neuropathy phenotypes, including motor behavioural deficits, reduced motor NCV and reduced motor axon calibre.^31^ To elevate tRNA^Tyr^ expression levels in *Yars1^E196K/E196K^* mice, we designed constructs for expression of tRNA^Tyr^ or tRNA^Gly-GCC^ driven by the mouse U6 promoter, an RNA polymerase III promoter. Two independent U6-tRNA^Tyr^ constructs were designed, either with or without the endogenous intron. Indeed, while intron splicing involves an additional processing step, the tRNA^Tyr^ intron was reported to be crucial for modification and stabilization of mature tRNA^Tyr^ in yeast.^34^ To validate the tRNA^Tyr^ expression constructs, we transfected them in HEK 293T cells and determined tRNA^Tyr^ levels after 24 h. Northern blotting revealed a significant upregulation of tRNA^Tyr^ levels for both constructs, by ∼4- to 5-fold (Fig. 5A and B).

**Figure 5:**
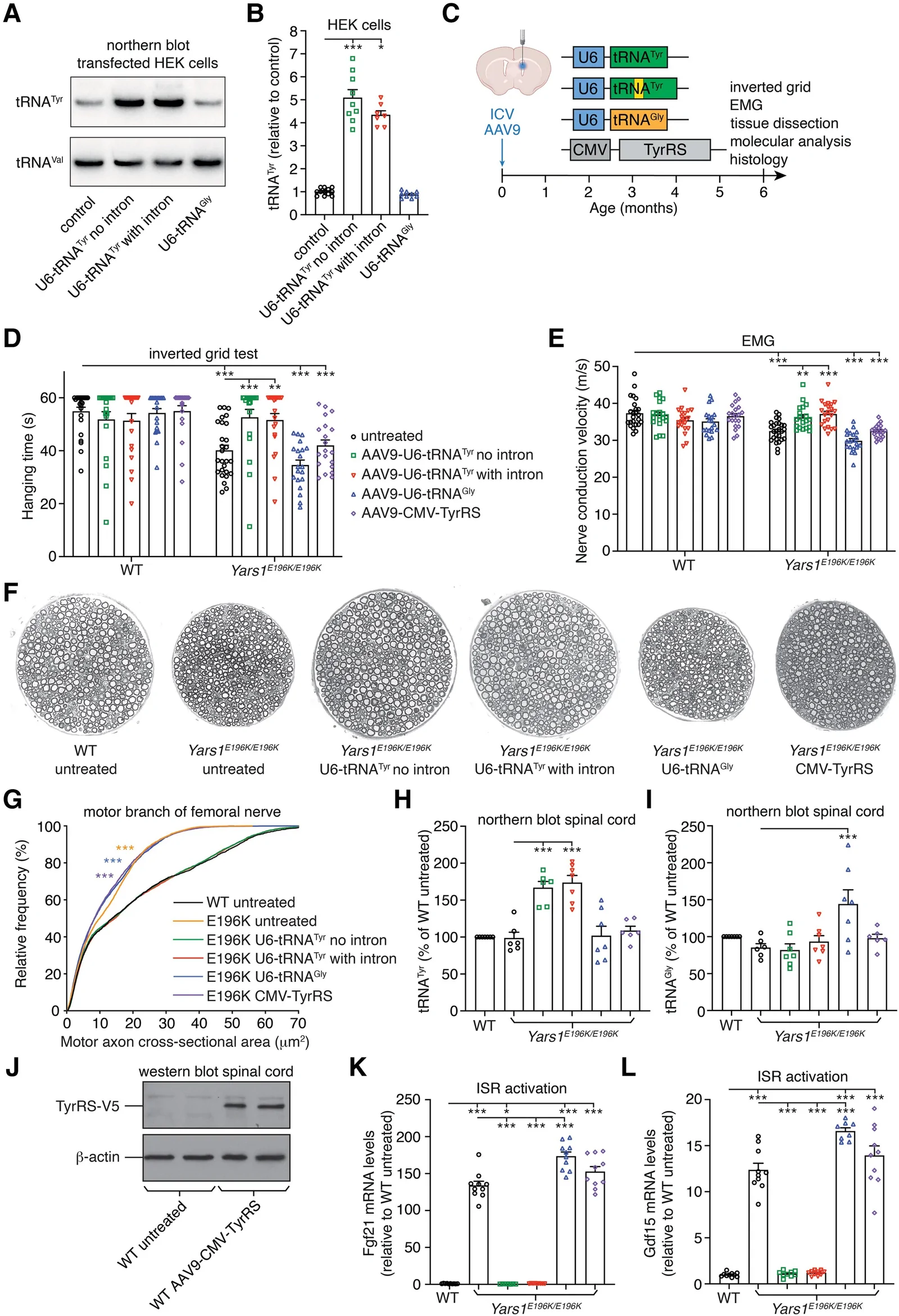
**tRNA^Tyr^ overexpression prevents peripheral neuropathy and integrated stress response activation in *Yars1^E196K/E196K^* mice**. (**A** and **B**) Representative images (**A**) and quantification (**B**) of northern blot to evaluate tRNA^Tyr^ levels in RNA extracts from HEK 293T cells transfected with U6-tRNA^Tyr^ (with or without intron), U6-tRNA^Gly^ or CMV-GFP (control) plasmid. tRNA^Val^ was used as a loading control, obtained after stripping and re-probing the blot with a tRNA^Val^ probe. *n* = 7–12 independent transfections per plasmid; *P < 0.05, ***P < 0.0005 by Kruskal–Wallis test with Dunn’s multiple comparisons test. (**C**) Experimental design to evaluate the therapeutic effect of ICV-administered AAV9-U6-tRNA^Tyr^ to newborn *Yars1^E196K/E196K^* mice. (**D** and **E**) Hanging time in the inverted grid test (**D**) and motor nerve conduction velocity as evaluated by EMG (**E**) of 6-month-old *Yars1^E196K/E196K^* versus wild-type (WT) littermate control mice, either untreated or treated with AAV9-U6-tRNA^Tyr^ (with or without intron), AAV9-U6-tRNA^Gly^ or AAV9-CMV-TyrRS (1.75 × 10^11^ vg/animal). *n* = 19–27 mice per experimental group, **P < 0.01, ***P < 0.0001 by two-way ANOVA with Tukey’s multiple comparisons test. (**F** and **G**) Representative images of semi-thin sections through the motor branch of the femoral nerve (**F**) and cumulative frequency of the cross-sectional area of individual axons (**G**) of WT or *Yars1^E196K/E196K^* mice, either untreated or treated with AAV9-U6-tRNA^Tyr^ (with or without intron), AAV9-U6-tRNA^Gly^ or AAV9-CMV-TyrRS. ***P < 0.0001 by the Kolmogorov–Smirnov test. (**H** and **I**) Quantification of northern blots to evaluate tRNA^Tyr^ (**H**) or tRNA^Gly^ (**I**) levels in the spinal cord of WT or *Yars1^E196K/E196K^* mice, either untreated or treated with AAV9-U6-tRNA^Tyr^ (with or without intron), AAV9-U6-tRNA^Gly^ or AAV9-CMV-TyrRS. *n* = 6–7 mice per experimental group; ***P < 0.005 by ordinary one-way ANOVA with Sidak’s multiple comparisons test. (**J**) Western blot to evaluate the expression of V5-tagged TyrRS in the spinal cord of WT mice, either untreated (*left two lanes*) or treated with AAV9-CMV-TyrRS (*right two lanes*). β-actin was used as a loading control. (**K** and **L**) mRNA levels of ATF4 target genes *Fgf21* (**K**) and *Gdf15* (**L**) as determined by qPCR on spinal cord extracts from 6-month-old *Yars1^E196K/E196K^* mice, either untreated or treated with AAV9-U6-tRNA^Tyr^ (with or without intron), AAV9-U6-tRNA^Gly^ or AAV9-CMV-TyrRS. Untreated WT littermate mice were used as a control. *n* = 7–10 mice per experimental group, *P < 0.05, ***P < 0.005 by Brown–Forsythe and Welch ANOVA with Dunnett’s T3 multiple comparisons test. ICV=intracerebroventricular; ISR=integrated stress response; TyrRS=tyrosyl-tRNA synthetase.

We therefore packaged both U6-tRNA^Tyr^ and the tRNA^Gly^ constructs in AAV9 capsid and delivered the AAV9 vectors via ICV injection to newborn *Yars1^E196K/E196K^* mice or WT littermate control mice, because this delivery paradigm results in efficient transduction of motor neurons throughout the spinal cord and sensory neurons in dorsal root ganglia, effectively targeting the cell types of interest.^35,36^ We also included an AAV9-CMV-TyrRS vector for expression of human WT TyrRS, to test for possible loss-of-function mechanisms contributing to CMT-*YARS1.*^5^ After AAV9 delivery, mice were aged and motor performance, motor NCV and motor axonal calibre were evaluated at 6 months of age (Fig. 5C). As expected, untreated *Yars1^E196K/E196K^* mice displayed a significantly reduced hanging time in the inverted grid test (Fig. 5D), reduced sciatic motor NCV (Fig. 5E) and reduced motor axonal cross-sectional area (Fig. 5F and G). In contrast, delivery of AAV9-U6-tRNA^Tyr^ vectors, either with or without intron, induced a full rescue of the impaired motor performance of *Yars1^E196K/E196K^* mice (Fig. 5D), and normalized their motor NCV (Fig. 5E) and motor axonal cross-sectional area (Fig. 5F and G). Treatment with AAV9-U6-tRNA^Gly^ vector did not rescue the motor performance defect, reduced NCV and reduced axon calibre of *Yars1^E196K/E196K^* mice (Fig. 5D–G), confirming that the phenotypic rescue is specific to tRNA^Tyr^ overexpression. Similarly, treatment with AAV9-CMV-TyrRS vector did not rescue the peripheral neuropathy phenotypes of *Yars1^E196K/E196K^* mice (Fig. 5D–G), indicating that these phenotypes are not attributable to a loss-of-function mechanism.

Northern blotting confirmed tRNA^Tyr^ overexpression in the spinal cord of *Yars1^E196K/E196K^* mice treated with AAV9-U6-tRNA^Tyr^ vectors by ∼70% (Fig. 5H), as well as tRNA^Gly^ overexpression in the spinal cord of *Yars1^E196K/E196K^* mice treated with AAV9-U6-tRNA^Gly^ (Fig. 5I). Similarly, expression of WT human TyrRS in the spinal cord of AAV9-CMV-TyrRS-treated mice was confirmed by western blot (Fig. 5J). Finally, treatment of *Yars1^E196K/E196K^* mice with AAV9-U6-tRNA^Tyr^ vectors fully prevented ISR activation in spinal motor neurons (Fig. 5K and L). In contrast, AAV9-U6-tRNA^Gly^ or AAV9-CMV-TyrRS treatment did not reduce expression of ATF4 target genes in *Yars1^E196K/E196K^* mice (Fig. 5K and L). As expected, treatment of WT littermate control mice did not activate the ISR or affect axonal calibre in the motor branch of the femoral nerve, irrespective of the AAV9 vector they received (Supplementary Fig. 1A–C). In all, our data are consistent with sequestration of tRNA^Tyr^ by CMT-mutant TyrRS as the molecular root cause of CMT-*YARS1*. Furthermore, AAV-tRNA^Tyr^ gene therapy can be explored as a novel therapeutic approach for CMT-*YARS1*.

## Discussion

Two decades ago, mono-allelic mutations in *YARS1* were identified as the genetic cause of CMT-*YARS1* (DI-CMTC), and partial loss of aminoacylation activity was initially suggested as a pathogenic mechanism.^15^ However, soon afterwards the TyrRS-E196K variant was shown to retain aminoacylation activity,^19,20^ indicating that loss of aminoacylation activity is not required to cause CMT-*YARS1*. Nevertheless, each of three CMT-TyrRS variants—including the aminoacylation-active TyrRS-E196K—substantially inhibited *de novo* protein synthesis in *Drosophila* motor and sensory neurons, suggesting mRNA translation defects as a unifying pathogenic mechanism underlying CMT-*YARS1.*^10^ Here, we identify tRNA^Tyr^ sequestration as the molecular mechanism underlying CMT-*YARS1*. Our findings that tRNA^Tyr^ overexpression rescues peripheral neuropathy and defective protein synthesis in *Drosophila* and mouse CMT-*YARS1* models and that CMT-TyrRS variants display stronger tRNA^Tyr^ association and induce selective ribosome pausing on tyrosine codons indicate that CMT-*YARS1* and CMT-*GARS1* share the same pathogenic mechanism. Specifically, the sequestration of the cognate tRNA(s) by the CMT-mutant aaRS leads to depletion of the cellular pool of this tRNA. Consequently, insufficient aminoacyl-tRNA production and supply to the ribosome increases ribosomal dwelling at the affected codons, which triggers general control nonderepressible 2 (GCN2)-mediated activation of the integrated stress response (this study and ^11,13^). Thus, a similar pathogenic mechanism may also underlie the other CMT subtypes linked to mutations in cytosolic tRNA synthetases, including alanyl-tRNA synthetase, tryptophanyl-tRNA synthetase, histidyl-tRNA synthetase, seryl-tRNA synthetase, aspartyl-tRNA synthetase and asparaginyl-tRNA synthetase, although this remains to be demonstrated. Our finding that elevating tRNA^Tyr^ levels fully prevents peripheral neuropathy, protein synthesis defects and ISR activation in *Drosophila* and mouse CMT-*YARS1* models indicates that previously suggested pathogenic mechanisms^16-18^ are either downstream of mRNA translation defects and ISR activation, or are secondary effects of peripheral neuropathy and not causal mechanisms. Further research is needed to distinguish between these possibilities.

We show for the first time that exogenous administration of tRNA by AAV9-mediated gene transfer induces a therapeutic effect in a CMT-aaRS mouse model. Indeed, AAV9-tRNA^Tyr^ administration to newborn, presymptomatic *Yars1^E196K/E196K^* mice fully prevented peripheral neuropathy and ISR activation. The presence of the endogenous intron in tRNA^Tyr^ was not required for the therapeutic effect, indicating that in mammals, dissimilar to yeast,^34^ expression of an intron-less tRNA^Tyr^ has no major impact on tRNA stability and functionality. To confirm the therapeutic potential of AAV-tRNA^Tyr^ gene therapy, the therapeutic effect of AAV-tRNA^Tyr^ vectors administered to symptomatic *Yars1^E196K/E19K^* mice should be evaluated. Nevertheless, our data indicate that elevating tRNA^Tyr^ levels may constitute a novel therapeutic approach for CMT-*YARS1*, similar to elevating tRNA^Gly^ levels for the treatment of CMT-*GARS1*.

## Supplementary Material

awag204_Supplementary_Data

## Data Availability

Ribosome profiling data were deposited in the Gene Omnibus (GEO) under the accession number GSE268213. Any newly generated materials, including DNA constructs and fly lines reported in this paper, are available from the corresponding author (erik.storkebaum@donders.ru.nl) upon request.
