# Supplementary material for: Tyrosyl-tRNA sequestration triggers tyrosyl-tRNA synthetase-associated peripheral neuropathy
Source: Brain. 2026 Aug 12;149(11):4052–65. doi: 10.1093/brain/awag204 (PMC13730582; doi:10.1093/brain/awag204)
Supplement: Supplementary file 1 [file awag204_supplementary_data.zip › brain-2026-00423-File007.pdf]

Tyrosyl-tRNA sequestration triggers tyrosyl-tRNA synthetase-associated peripheral neuropathy

Moushami Mallik,[1,†] Abigail L.D. Tadenev,[2,†] Natalia Mora,[1,†] Vanessa Lewandowski,[3] Daniel Köster,[3] María Landínez-Macías,[1] Robin Thompson,[3] María P. Menafra,[1] Noah K. Taylor,[4] Pascal van Lith,[1] Nick van Bakel,[1] Marica Catinozzi,[1] Scott Q. Harper,[4,5] Zoya Ignatova,[3] Robert W. Burgess,[2] Erik Storkebaum,[1]

[†]These authors contributed equally to this work.

[1]Molecular Neurobiology Laboratory, Donders Institute for Brain, Cognition and Behaviour and Faculty of Science, Radboud University, Nijmegen, Netherlands

[2]The Jackson Laboratory, Bar Harbor, ME, USA.

[3]Biochemistry and Molecular Biology, Department of Chemistry, University of Hamburg, Hamburg, Germany

[4]Jerry R. Mendell Center for Gene Therapy, The Abigail Wexner Research Institute at Nationwide Children's Hospital, Columbus, OH, USA

[5] Department of Pediatrics, The Ohio State University College of Medicine, Columbus, OH

Corresponding author: Erik Storkebaum

Donders Center for Neuroscience, Radboud University

Heyendaalseweg 135

6525 HR Nijmegen, The Netherlands

erik.storkebaum@donders.ru.nl

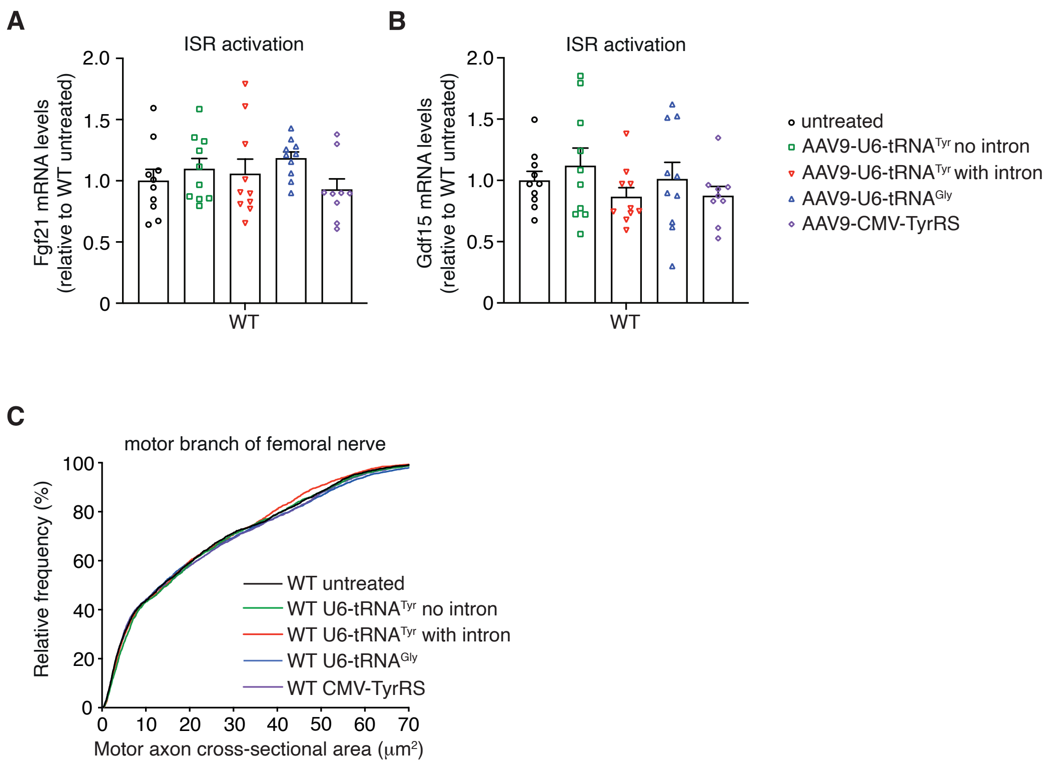

Figure S1: AAV-tRNA^{Tyr} and AAV-TyrRS treatments do not induce the ISR or affect axon caliber in WT mice. **A,B,** mRNA levels of ATF4 target genes Fgf21 (A) and Gdf15 (B) as determined by qPCR on spinal cord extracts from 6-month-old WT control mice, either untreated or treated with AAV9-U6-tRNA^{Tyr} (with or without intron), AAV9-U6-tRNA^{Gly}, or AAV9-CMV-TyrRS. The untreated WT data are the same as in Figure 4K,L. *n*=9-10 mice per experimental group, p=not significant by one-way ANOVA with Dunnett's multiple comparisons test. **C,** Cumulative frequency of the cross-sectional area of individual axons in the motor branch of the femoral nerve of WT mice, either untreated or treated with AAV9-U6-tRNA^{Tyr} (with or without intron), AAV9-U6-tRNA^{Gly}, or AAV9-CMV-TyrRS. p=not significant by Kolmogorov-Smirnov test.

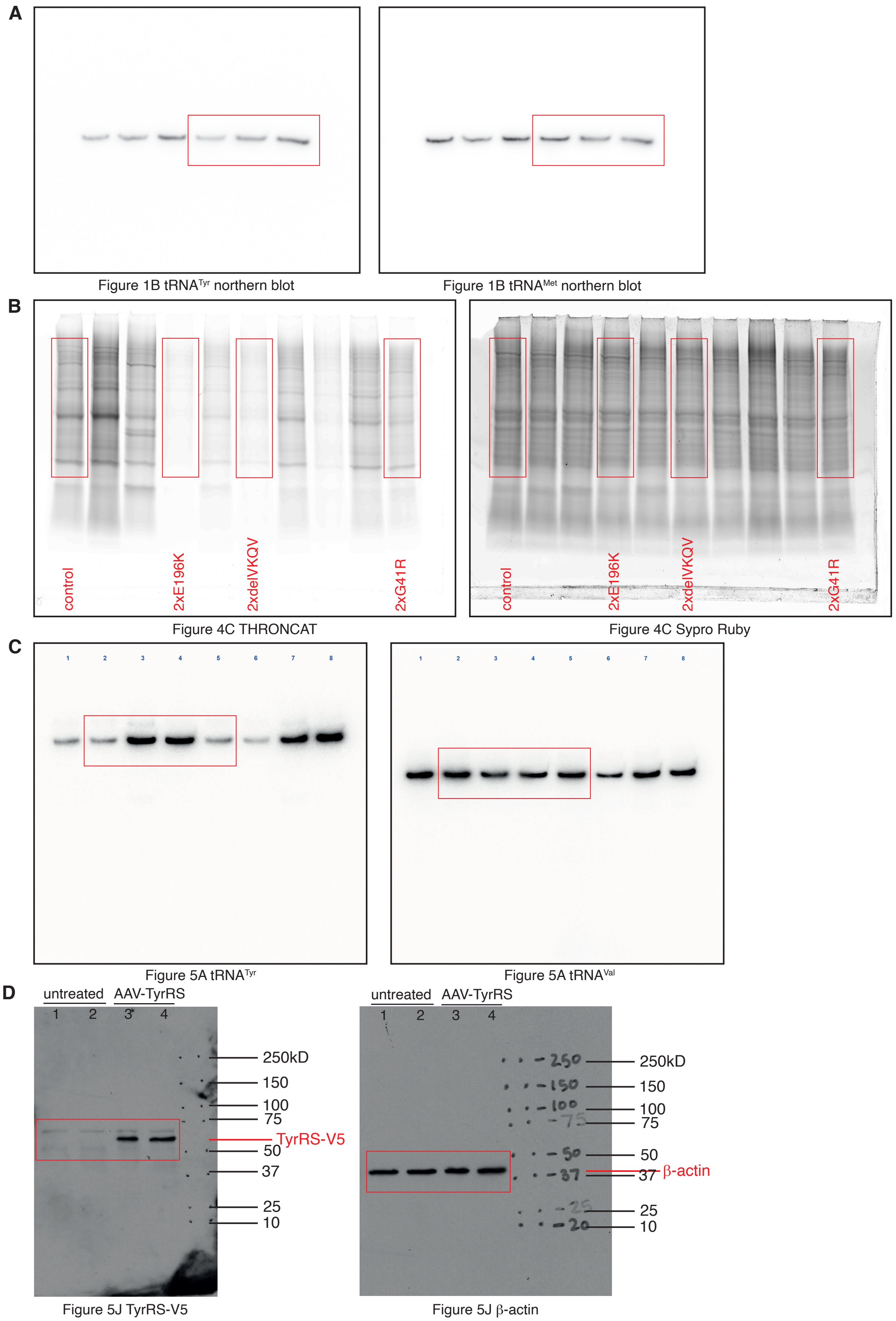

Figure S2: Full-length blot images. **A-D,** Full-length images of blots shown in Figure 1B (A), 4C (B), 5A (C), and 5J (D). The parts of the images shown in the main figure panels are

delineated by a red square. For the northern blots shown in figure 5A, the images were flipped horizontally in order to show the lanes in the logical order.

Supplementary Materials and Methods

Analysis of sensory neuron dendritic morphology

Class IV multidendritic (MD) sensory neuron morphology imaging was performed on living third instar larvae. Microscopy slides were prepared with two coverslips glued on as spacers with conventional superglue. Larvae were thoroughly washed in tap water and a single chilled larva was immobilized under a coverslip, which itself was glued onto the spacing coverslips to maintain equal spacing and pressure on the larvae. Only dorsal class IV MD neurons of larval segment A1 were imaged using a Leica SP8 laser scanning confocal microscope. For quantification, maximum intensity projections were processed with Adobe Photoshop, rotated and digitally placed under a transparent grid containing a crosshair. The crosshair was used to fit the grid onto the cell body of the class IV MD neuron in order to divide the dendritic tile into four quadrants. The posteromedial quadrant of the imaged da neuron was chosen for quantification of dendritic coverage, because imaging artifacts and interference of denticle bands were minimal in that area. For quantification within the posteromedial quadrant, a sector of 200 μm to the medial and 280 μm to the posterior was defined, equaling 56,000 μm^2 divided into 560 squares of 8 x 8 pixels each. Presence versus absence of a piece of dendrite in the defined boxes was scored and calculated as measure of relative dendritic coverage.

Automated negative geotaxis assay to analyze motor performance

To assay motor performance, newly eclosed male flies were collected and divided into groups of 10 individuals. Until measurement, flies were maintained at 25°C with a 12-h light/dark cycle on standard cornmeal medium. 7-day-old flies were evaluated in a rapid iterative negative geotaxis assay (RING-assay), which was previously established in the Storkebaum lab.[10] The assay is based on the innate escape response of flies to climb up the wall of a vial after being tapped down to its bottom. Flies were transferred into test tubes without anesthesia and three

iterative measurements of groups of 10 flies per test tube were video recorded with a Nikon D3100 DSLR camera. The resulting movies were converted into 8-bit grayscale TIF image sequences with 10 frames/s. Subsequently, image sequences were analyzed using an MTrack3 plug-in that automatically imports images in ImageJ, subtracts backgrounds, and filters and binarizes images to allow tracking of flies. Average climbing speed (mm/s) of all tracked flies was determined, averaged per test tube, and compared between genotypes.

Construct	Cloning Primers	Primer sequence
Drosophila tRNA$^{Tyr\text{-}GTA}$	vector_fr_1_fwd	tctagaGGTCACTATCAGTCAAAATAAAAT
	vector_fr_1_rev	ggtaccCAAAGGCGGTAATACGGTTATC
	tRNA_Tyr_fr_2_fwd	taaccgtattaccgcctttgggtaccGGATCTGCATATTTATTTGTATTTG
	tRNA_Tyr_fr_2_rev	tatttatgcgGTTGCACAACAAAACTCG
	tRNA_Tyr_fr_3_fwd	gttgtgcaacCGCATAAATAAATAAATTAAATAAATAAATTATTG
	tRNA_Tyr_fr_3_rev	tcgacttgtgCTTTGAAATGCGAATGTAAATTG
	tRNA_Tyr_fr_4_fwd	catttcaaagCACAAGTCGAAGAAAGTTTTG
	tRNA_Tyr_fr_4_rev	tattttgactgatagtgacctctagaCCAGATCTTTCAGATCCTG
	**Sequencing Primers**	**Primer sequence**
	pEntr_seq_FW	CCTTTTTACGGTTCCTGGCC
	pEntr_seq_REV	TGCCAGGAAACAGCTATGACC
	Tyr_f2-3_seq_FW	CGAGTTTTGTTGTGCAACCGC
	Tyr_f2-3_seq_RV	TAATTTATTTATTTATGCGGTTGCACAAC
	Tyr_f3-4_seq_FW	TCGCATTTCAAAGCACAAGTCG
	Tyr_f3-4_seq_RV	CGACTTGTGCTTTGAAATGCGA

Table S1.

Sequences of primers used for PCR and sequencing of the indicated transgenic construct.

**Species**	**probe target**	**probe sequence (5'➔3')**
Drosophila	Dm_tRNA_Tyr^{GTA}	TCCTTCGAGCCGGATTTGAACC
Drosophila	Dm_tRNA_Met^{CAT}	TGGTGGAGATGCGGGGTATCGA
Mouse	Mm_tRNA_Tyr^{GTA}	TCCTTCGAGCCGGATTTGAACC
Mouse	Mm_tRNA_Gly^{GCC}	GGCGAGAATTCTACCACTGAACCACCAA
Mouse	Mm_tRNA_Val^{CAC}	AGGCGAACGTGATAACCACTACACTACGGA

Table S2.

Sequence and tRNA target of probes used for northern blotting.

**Mouse gene**	**forward primer**	**reverse primer**
Fgf21	CTGCTGGGGGTCTACCAAG	CTGCGCCTACCACTGTTCC
Gdf15	GAGCTACGGGGTCGCTTC	GGGACCCCAATCTCACCT
β-actin	GATCTGGCACCACACCTTCT	GGGGTGTTGAAGGTCTCAAA

Table S3.

Target genes and primer sequences used for qRT-PCR.
