# Supplementary material for: Tyrosyl-tRNA sequestration triggers tyrosyl-tRNA synthetase-associated peripheral neuropathy
Source: Brain. 2026 Aug 12;149(11):4052–65. doi: 10.1093/brain/awag204 (PMC13730582; doi:10.1093/brain/awag204)
Supplement: Supplementary file 1 [file awag204_supplementary_data.zip › brain-2026-00423-File008.pdf]

Author contributions

M.M., A.L.D.T., N.M., V.L., M.L.M., R.T., M.P.M., N.K.T., P.v.L., N.v.B., and M.C. performed experiments. D.K. and Z.I. analyzed the ribosome profiling data. All authors analyzed and discussed the data. E.S. conceived and initiated the project. E.S., R.W.B. and Z.I. supervised the project. E.S. wrote the manuscript with contribution of M.M., A.L.D.T and N.M. All authors contributed to the experimental design and interpretation and commented on the manuscript. M.M., A.L.D.T., and N.M. are co-first authors because each of them made an essential and substantial contribution to the paper.
